# High-resolution MALDI mass spectrometry imaging of gallotannins and monoterpene glucosides in the root of *Paeonia lactiflora*

**DOI:** 10.1038/srep36074

**Published:** 2016-10-31

**Authors:** Bin Li, Dhaka Ram Bhandari, Andreas Römpp, Bernhard Spengler

**Affiliations:** 1 Institute of Inorganic and Analytical Chemistry, Justus Liebig University Giessen, Heinrich-Buff-Ring 17, 35392 Giessen, Germany; 2 Department of Pharmacy, University of Copenhagen, Universitetsparken 2, 2100, Copenhagen, Denmark

## Abstract

High-resolution atmospheric-pressure scanning microprobe matrix-assisted laser desorption/ionization mass spectrometry imaging (AP-SMALDI MSI) at 10 μm pixel size was performed to unravel the spatio-chemical distribution of major secondary metabolites in the root of *Paeonia lactiflora*. The spatial distributions of two major classes of bioactive components, gallotannins and monoterpene glucosides, were investigated and visualized at the cellular level in tissue sections of *P. lactiflora* roots. Accordingly, other primary and secondary metabolites were imaged, including amino acids, carbohydrates, lipids and monoterpenes, indicating the capability of untargeted localization of metabolites by using high-resolution MSI platform. The employed AP-SMALDI MSI system provides significant technological advancement in the visualization of individual molecular species at the cellular level. In contrast to previous histochemical studies of tannins using unspecific staining reagents, individual gallotannin species were accurately localized and unequivocally discriminated from other phenolic components in the root tissues. High-quality ion images were obtained, providing significant clues for understanding the biosynthetic pathway of gallotannins and monoterpene glucosides and possibly helping to decipher the role of tannins in xylem cells differentiation and in the defence mechanisms of plants, as well as to investigate the interrelationship between tannins and lignins.

Plant tissues can be thought of as highly organized chemical factories where diverse secondary metabolites are produced, transported and accumulated in specific compartments. Generally the roles of secondary metabolites in plants are considered to provide protection – either against other organisms such as herbivores and pathogenic bacteria or against exposure to UV light – to function as a pollination attractant, and even to provide a means for chemical communication with the surrounding environment^1^. Moreover, thousands of natural products have been isolated from plants and many have been shown to possess significant physiological effects upon human health and disease and are commonly used as herbal extracts and herbal medicines. Due to the prominent effect that secondary metabolites have on plants and humans, several analytical techniques have been used to identify their chemical structures, quantify contents and even map their spatial distribution^2^. While it is well-known that visualization of tissue/cell-specific localization of secondary metabolites provides straightforward clues to decipher their functions, techniques for this aim meet several challenges and are still in the developmental phase^3^,^4^. This is especially the case when comparing methods of secondary metabolite visualization to well-established approaches for localization of mRNA and proteins such as green fluorescent protein and protein-specific antibodies that are commonly used for the high-throughput determination of subcellular localization of macromolecules^5^. Traditional histochemical staining or immunohistochemical (IMH) approaches have been used for localization of small molecules, although these techniques are more adeptly applied to the localization of macromolecules. Intrinsic limitations of existing methods are either the inability to distinguish individual metabolites or the inherent loss/delocalization of small molecules that arises during rinsing and fixation steps of a typical staining process^6^.

Mass spectrometry imaging (MSI) has emerged over the past two decades to become a fundamental tool for label-free, untargeted spatio-chemical characterization of biological systems^7^. Owing to its unparalleled capabilities, a correlation of histological features with individual metabolites can go beyond the level of classical histochemical staining. In the field of MSI, matrix-assisted laser desorption/ionization (MALDI) imaging, introduced in the mid 1990’s^8^, is the most extensively used MSI technique for molecular imaging of both mammalian^9^ and plant tissues^10^. Besides MALDI, secondary ion mass spectrometry (SIMS)^11^ and various emerging ambient ionization techniques^12^, such as desorption electrospray ionization (DESI)^13^ and laser ablation electrospray ionization (LAESI)^14^, are the other popular sampling probes that have been employed in MS-based imaging measurements. SIMS imaging offers the highest spatial resolution among MSI techniques, down to tens of nanometers. However, major limitations of SIMS, including strong fragmentation of molecular ions and a relatively low signal intensity in the high mass range (*m/z* > 1000), have precluded its widespread use. DESI imaging is characterized by minimal sample preparation, but the spatial resolution is restricted to about 100 μm in routine analysis due to the difficulty in focusing charged droplets during spray^15^,^16^.

Also MALDI imaging experiments were performed for the localization of secondary metabolites in plant tissues, and previous approaches for mapping the distribution of plant metabolites were confined to 50–200 μm spatial resolution^17^. For plant tissues with numerous well-defined compartments, improvement in spatial resolution down to the cellular level provides access to a much greater chemical detail and therefore holds great potential to reveal the functions of tissue/cell-specific metabolites. For instance, asymmetric metabolic features in the cross section of maize leaf were mapped at 5 μm spatial resolution with an oversampling method^18^; C_29_ alkane distribution on surface of single pollen grains of *Arabidopsis* was resolved at ~12 μm spatial resolution^19^; as well as various metabolite distributions on intact seedling of *A. thaliana* were visualized below 10 μm spatial resolution^20^. Likewise, tissue-specific localization of free flavonoids in rhizome of liquorice was visualized in the cork layer with 10 μm laser pixel resolution^6^ and at 5 μm pixel resolution metabolites in wheat seed were visualized^21^. Recently, cell-specific localization of terpenoid indole alkaloids in the stem tissues of *Catharanthus roseus* was demonstrated with MALDI imaging and single-cell MS^22^. High spatial resolution alone is not sufficient to generate high quality ion images. The quality of an image also depends on high spectral resolution and high mass accuracy, with which nearly isobaric ions can be resolved, facilitating compound identification through a rapid search in online databases such as METLIN^23^. Fourier transform ion cyclotron resonance (FT-ICR) and orbital trapping mass spectrometers are ultra-high resolution mass analysers, offering a mass resolving power greater than 100,000 and mass accuracies of better than 1 ppm. In this respect, our group recently introduced a high-performance atmospheric-pressure scanning microprobe MALDI (AP-SMALDI) ion source^24^ in which the laser spot size can be focused down to 5 μm, allowing for a pixel size down to 3 μm in diameter and enabling localization of metabolites at the single cell level^6^,^25^. Furthermore, coupling to an orbital trapping mass spectrometer allows for high mass resolution and high mass accuracy, ideal for high throughput metabolite profiling^26^.

The root of *Paeonia lactiflora* (peony), a well-known traditional Chinese medicine, has been used for more than 1200 years in the treatment of various human diseases such as arthritis and dysmenorrhea^27^,^28^. As shown in Fig. 1, major components isolated from *P. lactiflora* root include monoterpene glucosides (MGs) and gallotannins^29^. Although pharmacological activities and chemical composition of major secondary metabolites isolated from *Paeonia* species have been extensively investigated, their spatial distribution in tissue are scarcely reported, in particular for gallotannins, which are a subclass of hydrolysable tannins. Tannins are known as a vital class of secondary metabolites in plant chemical defence mechanisms used for protection against pathogens, insects and herbivores^30^. A few histochemical staining methods have been applied for the visualization of the distribution of various tannins^31^. However, it should be noted that these staining methods are unable to distinguish tannins from other polyphenolic components since indicators are unspecific (based on all substrates having phenolic groups).

In the present work, spatio-chemical information on the distribution of metabolites in the root of *P. lactiflora* was explored by the combination of a high spatial-resolution of 10 μm and a high mass resolution of 140,000 at *m/z* 200. Due to the high quality of the obtained ion images, spatial contexts of individual gallotannins and monoterpenes could be revealed for the first time at the cellular level. High mass accuracy (<3 ppm root mean square error (RMSE)) and on-tissue MS/MS measurements helped in identifying metabolites. These results are in accordance with earlier histochemical studies conducted on different plant organs^32^,^33^,^34^, but are more detailed. Precise spatial information presented here provides significant improvement of our understanding of several key issues, regarding biosynthesis pathways as well as the deposition and transportation of tannins and monoterpenes.

## Results

A representative single-pixel mass spectrum obtained from the root cross-section of *P. lactiflora* is shown in Fig. 2. Two major specific classes of secondary metabolites i.e. gallotannins and monoterpene glucosides (MGs), were detected as ionic adducts with sodium and potassium. Owing to the high mass resolution, it was possible to distinguish peaks with mass differences as small as of 0.02 u as shown in Fig. 2a. The identification of metabolites was based on accurate mass and/or on-tissue MS/MS measurements. At very low ion abundances, no MS/MS data but still the accurate mass of Na^+^ and/or K^+^-attached ions was available, assisting the identification of the compounds (Figs S1–S3 and Table S1).

### Visualization of the distribution of gallotannins in the root cross-section of *P. lactiflora*

**Figure 1:**
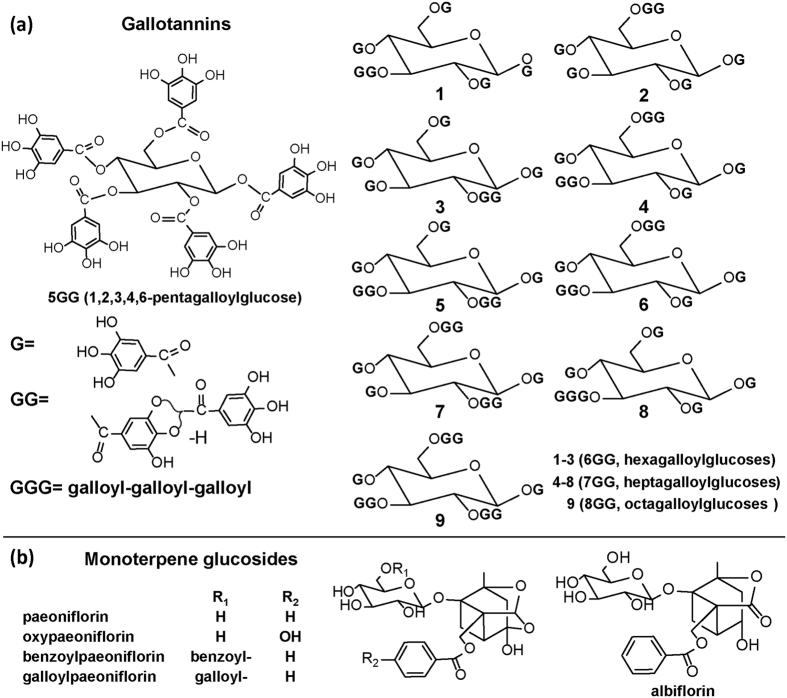
Chemical structures of selected metabolites from root extracts of *P. lactiflora*, (**a**) gallotannins and (**b**) monoterpene glucosides.

**Figure 2:**
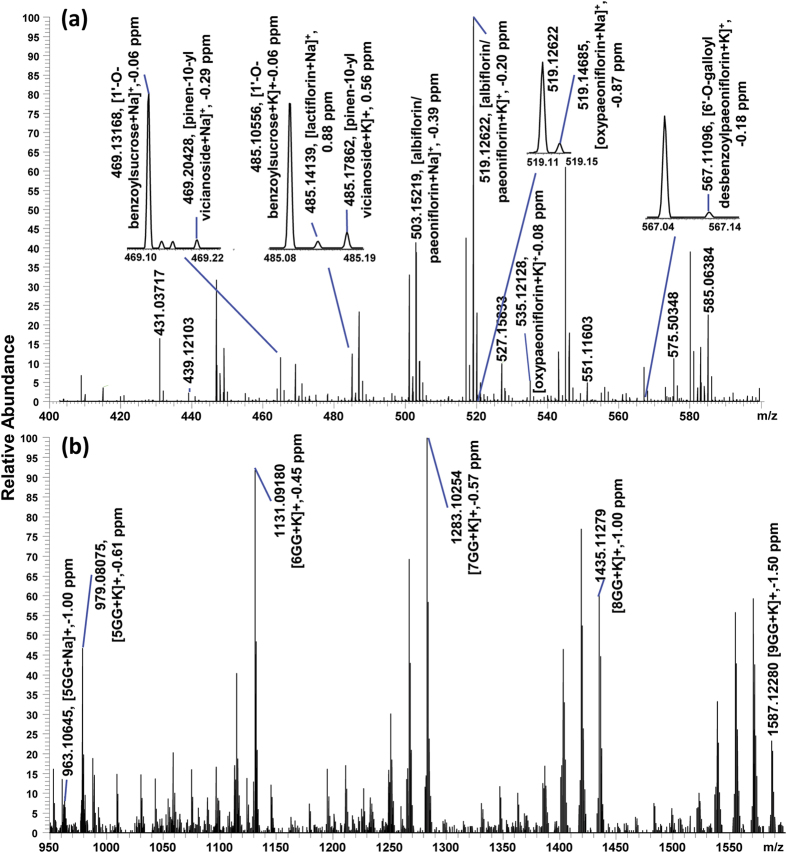
Mass spectrum acquired from a single 30 μm pixel for mass range *m/z* = 400–600 (**a**) and *m/z* = 950–1600 (**b**) from the root cross-section of *P. lactiflora*. Identified compounds are labeled with measured mass, compound name, and mass deviation. See Table S1 for more details.

**Figure 3:**
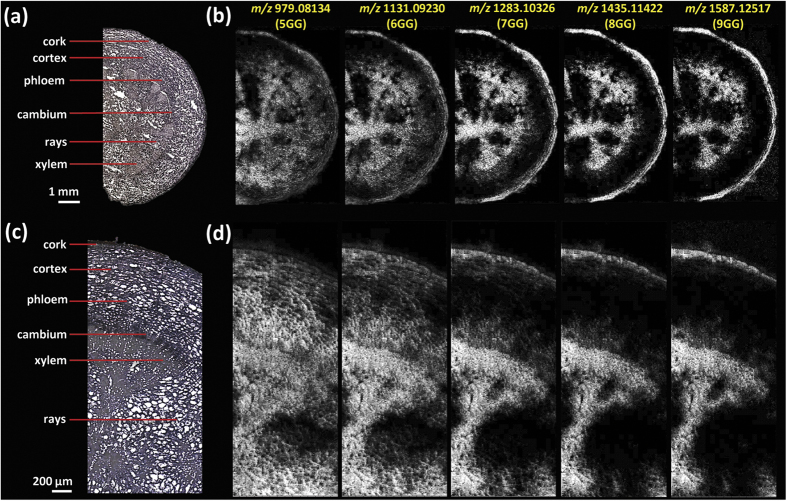
MALDI images of gallotannins in the *P. lactiflora* root, recorded with a scanning step size ( = pixel size) of 30 μm and 10 μm, respectively. (**a**) Optical image of the ½ root and (**c**) confined region of interest. (**b**) Ion images of gallotannins at 30 μm step size and 260 × 140 pixels per image, and at (**d**) 10 μm step size and 360 × 140 pixels per image. All ions are displayed using the same intensity scale (Gray: 0–255). The mass accuracy was better than 2 ppm (RMSE), and a bin width of *m/z* =  ± 5 ppm was used for image generation. Images represent the potassium adducts of the compounds listed in Table 1.

Figure 3 shows ion images of various gallotannins visualized in the root section with a pixel size of 30 μm and 10 μm, respectively. A number of gallotannins were detected in particular at the cork layer and the xylem region including pentagalloylglucose (5GG) (*m/z* = 979.08134, [M + K]^+^), hexagalloylglucose (6GG) (*m/z* = 1131.09230, [M + K]^+^), heptagalloylglucose (7GG) (*m/z* = 1283.10326, [M + K]^+^), octagalloylglucose (8GG) (*m/z* = 1435.11422, [M + K]^+^) and nonagalloylglucose (9GG) (*m/z* = 1587.12517, [M + K]^+^). RMSE values of K^+^ adduct ions were calculated to be less than 2.5 ppm for each ion (Table S1). *In situ* MS/MS measurements were conducted for compound identification, and the specific fragments obtained were consistent with previously reported MS/MS data acquired by electrospray ionization MS in the negative ion mode^35^. All gallotannin ions produced abundant characteristic fragment ions including [M + K/Na-170]^+^, [M + K/Na-170–152]^+^, and [M + K/Na-170-152-170]^+^, corresponding to successive or simultaneous loss of gallic acids (170 Da) and galloyl groups (152 Da) (Figure S1). It should be noted that, as shown in Fig. 1a, a number of isomeric gallotannins are present in peony root, which is a challenge in the structure identification of isomers by only using accurate mass and MS/MS.

Initially, a pixel size of 30 μm was used in order to obtain an overview of the metabolite distribution in the ½ root section from cork layer to the central region, representing the anatomical features of the root (Fig. 3a,b). To obtain fine localization of metabolites at the cellular level a higher spatial resolution with 10 μm pixel size was then used to acquire ion images in a confined area (Fig. 3c,d). The distribution patterns of 5GG, 6GG, 7GG, 8GG and 9GG exhibited both regional similarities and differences. In peony root, gallotannins were commonly deposited in the cork layer and xylem regions. However, there is a gradual decrease of gallotannin pixel coverage from 5GG to 9GG in the parenchyma cells of cortex and xylem regions. This is also obvious from Table 1, regarding the reduced value of pixel coverage in both 30 μm and 10 μm spatial resolution experiments.

### Visualization of the distribution of paeoniflorin and its derivatives in the root cross-section of *P. lactiflora*

**Figure 4:**
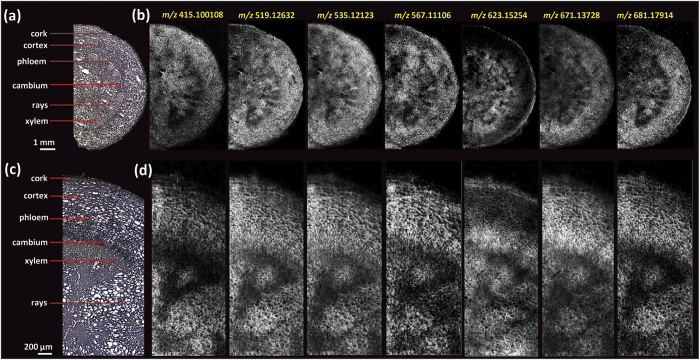
MALDI images of monoterpene glucosides in the *P. lactiflora* root, recorded with a scanning step size of 30 μm and 10 μm, respectively. (**a**) Optical image of the ½ root and (**c**) confined region of interest. (**b**) Ion images of monoterpene glucosides at 30 μm step size and 260 × 140 pixels per image, and at (**d**) 10 μm step size and 360 × 140 pixels per image. All ions are displayed using the same intensity scale (Gray: 0–255). The mass accuracy was better than 2 ppm (RMSE), and a bin width of *m/z* =  ± 5 ppm was used for image generation. Each individual image represents the K^+^ adducts of the compounds listed in Table 2.

Figure 4 shows ion images of various MGs visualized in the root section with a pixel size of 30 μm (Fig. 4b) and 10 μm (Fig. 4d), respectively. The distribution patterns of paeoniflorin and its derivatives present a high degree of similarity (Table S2). They were found mostly in the cork layer, cortex, cambium, and xylem rays. Each individual image represents putatively identified K^+^/Na^+^ adducts of paeoniflorin and its derivatives included in Table 2. Corresponding RMSE values of K^+^/Na^+^ adduct ions of the compounds were less than 1.5 ppm for each ion. Paeoniflorin (PA) and albiflorin (AL) are the two major components accumulated in amounts up to 3% in the root of *P. lactiflora*^36^. However, these two compounds are compositional isomers and therefore cannot be mass-discriminated even with ultra-high mass resolution. Both of them were detected as [M + Na]^+^ at *m/z* 503.15239 and [M + K]^+^ at *m/z* 519.12632 in positive-ion mode. On-tissue MS/MS experiments were conducted on [M + Na]^+^ but the common fragments obtained at *m/z* 381.1156 ([M-(benzoic acid) + Na]^+^), *m/z* 341.0996 ([M-glu + Na]^+^), *m/z* 219.0628 ([aglycone + Na]^+^) and *m/z* 185.0420 ([glu + Na]^+^) (Figure S1) made it impossible to distinguish them.

For the region of interest, a high spatial resolution imaging experiment was performed with a spatial resolution of 10 μm (Fig. 4d). Highly resolved images reveal detailed morphological and chemical features. In the xylem region, heterogeneous distributions of various MGs were visualized in a sector. Relatively low intensities were detected specifically in xylem vessels and fibres. In a small well-defined region of the xylem, MGs expressed high abundances, possibly pointing to different cell types in the xylem. Preliminary studies of MG distributions were only performed on the root tissues of *Paeonia moutan* (tree peony)^37^, which belongs to the same genus *Paeoniaceae* as *P. lactiflora* but classified as section *Moutan.* Results from quantitative thin layer chromatography (TLC) showed that four MGs including PA, benzoyl-PA, benzoyloxy-PA and oxy-PA, mainly exist in periderm and cortex regions, but have lower abundance in xylem parts. Therefore, results presented here indicate that the biosynthesis pathways of secondary metabolites are more likely to share similar features in deposition and transportation in the same generic plants.

### Visualization of the distribution of primary and other secondary metabolites in the root cross-section of *P. lactiflora*

**Figure 5:**
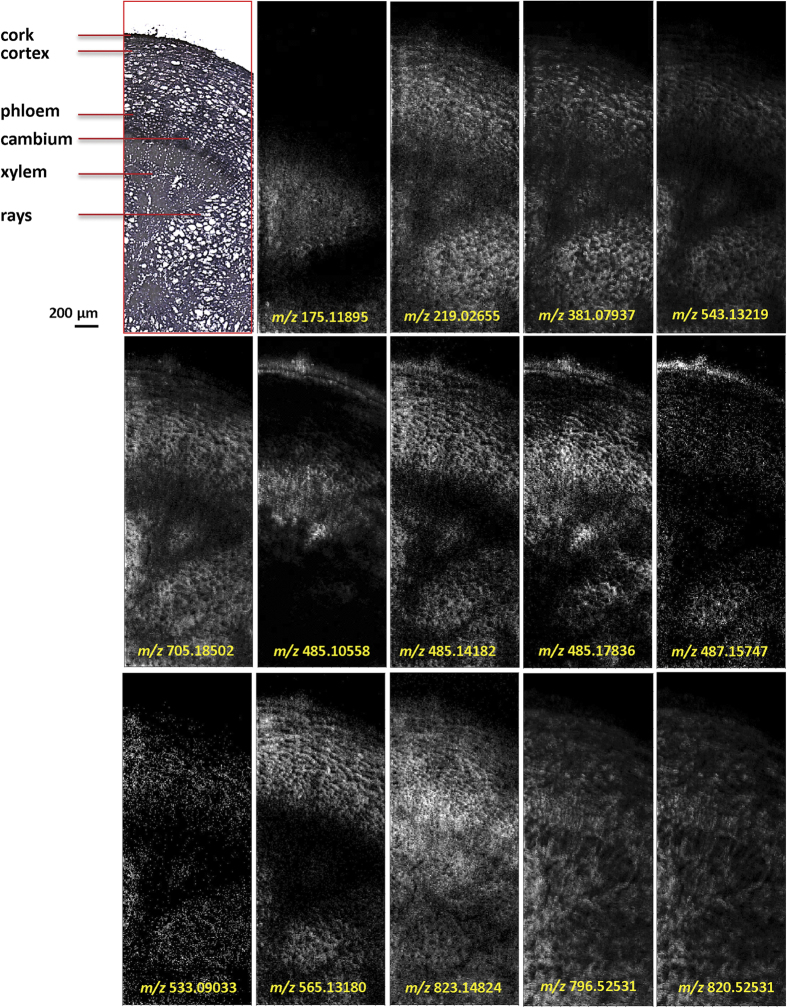
MALDI images of selected primary and other secondary metabolites in the *P. lactiflora* root, recorded with a spatial resolution of 10 μm and 360 × 140 pixels per image. All ions are displayed using the same intensity scale (Gray: 0–255). The mass accuracy was better than 1 ppm (RMSE), and a bin width of *m/z* =  ± 5 ppm was used. Each individual image represents the H^+^/Na^+^/ K^+^ adducts of the compounds included in Table 3.

Besides gallotannins and MGs, a number of additional ions, such as amino acids, carbohydrates, lipids and monoterpenes (Table 3) were putatively identified using our high resolution MSI platform. With a 10 μm spatial resolution, tissue-specific distributions of these metabolites were accurately visualized in the root (Fig. 5). For example, arginine (*m/z* 175.11895, [M + H]^+^) was found mainly in the xylem vessels and fibres, with homogenous distributions in the xylem, different from the cell-specific distributions of MGs and complex gallotannins, e.g. 8GG and 9GG (Fig. 3d). Additionally, monosaccharides (*m/z* 219.02655, [M + K]^+^), disaccharides (*m/z* 381.07937, [M + K]^+^), trisaccharides (*m/z* 543.13219, [M + K]^+^), as well as tetrasaccharides (*m/z* 705.18502, [M + K]^+^) were visualized and were found to have similar distribution patterns, with primary accumulation in the cortex and xylem rays.

With the benefit of high mass-resolving power three ions neighbouring within a narrow mass range of 0.1 mass units, were successfully resolved and putatively identified as [benzoylsucrose + K]^+^ (*m/z* 485.10558), [lactiflorin + Na]^+^ (*m/z* 485.14182) and [pinen-10-yl-vicianoside + K]^+^ (*m/z* 485.17836). As shown in Fig. 5, lactiflorin and pinen-10-yl-β-vicianoside show similar distribution patters as PA/AL (Fig. 4d). However, [benzoylsucrose + K]^+^ (*m/z* 485.10558) shows distinct deposition sites, in contrast to [benzoylpaeoniflorin + K]^+^ (*m/z* 623.15253) (Fig. 4d), mainly accumulating in the cork layer and cambium region. Each individual image in Fig. 5 represents either H^+^, Na^+^, or K^+^ adducts of the compounds, as listed in Table 3.

## Discussions

Unlike in well-established gene and protein analysis, deciphering the relationship between location and function of small molecules offers a great challenge, primarily due to metabolite transport between compartments and the complicated metabolic network that exists in both time and space^38^. With its unique ability to provide spatial information and chemical characterization, high resolution MALDI-MSI provides unparalleled chemical imaging beyond traditional staining methods, making it possible to map hundreds to thousands of components in the tissue sections of biological samples. MSI is therefore gradually gaining importance in plant science^4^,^17^. In the present work, the spatial distributions of species-specific secondary metabolites in the root of *P. lactiflora* were complementarily investigated at a cellular resolution for the first time, which is rather difficult to be explored with histochemical methods if no specific probes were designed for individual metabolites. MALDI imaging, however, is confined to approximately 5 μm spatial resolution, being a limitation for subcellular imaging. Additionally, localization of biosynthetic enzymes with MSI is extremely challenging due to their low abundance, degradation, and low desorption/ionization efficiencies of proteins from plant tissue sections. Therefore, microscopy-based imaging techniques are used to localize enzymes in cells and their sub-compartments. With both, high specificity and high lateral resolution of IMH methods, the deposition sites of gallotannins and ellagitannins and acyltransferase were investigated in young oak root tissues at subcellular level. It was revealed that parenchyma cell walls of cortex and endodermis cells are special accumulation sites^34^. However, gallotannins and ellagitannins were stained non-specifically. In contrast to other imaging techniques such as optical imaging, magnetic resonance imaging (MRI), X-ray computed tomography (CT) and positron emission tomography (PET), *in vivo* analysis is inaccessible to MALDI MSI^39^. Therefore, combining MSI techniques with various imaging modalities is becoming a topic of high interest to obtain more detailed spatio-chemical information on plant secondary metabolites and localization of associated biosynthetic enzymes, which will enhance the understanding of synthesis, transportation and accumulation of metabolites in plant tissues^40^.

A lateral resolution of 30 μm in the initial experiments was sufficient to resolve metabolite localizations, but detailed morphological features at the cellular level were unavailable (Figs 3b and 4b). Therefore, AP-MALDI imaging experiments with a higher lateral resolution of 10 μm were performed. As shown in Figs 3d and 4d, for example, cell populations in xylem rays with an approximate diameter of 30–70 μm were clearly resolved at 10 μm pixel size, well correlated to the histological question. Furthermore, subtle differences of metabolites resolved at 10 μm resolution could not be confidently justified at 30 μm resolution (Figure S4).

Current MS-based molecular imaging is mainly applied for targeted analysis, although it has great potential for global imaging of metabolites. High resolution in mass and space is a prerequisite, due to the high degree of complexity in biological tissue, whereas the identification of ions is a challenge for experiments performed with low or medium resolution mass spectrometers. Benefiting from the ultra-high mass resolving power and mass accuracy provided by orbital trapping mass spectrometers, closely neighbouring peaks with the same nominal mass (a fairly common issue in plant tissues) can be separated and the obtained accurate mass can be directly used for calculation of elemental composition and even for high fidelity mass spectra library searches. Therefore, small pixel size, high mass accuracy and mass resolution are necessarily a prerequisite for high-quality MS imaging of plant tissues.

**Figure 6:**
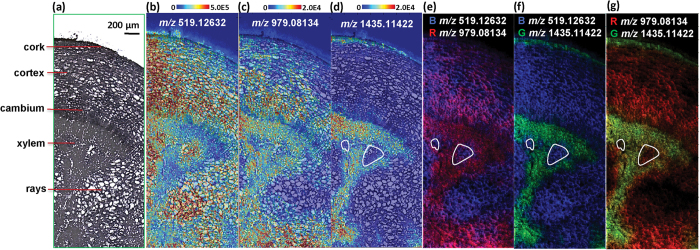
Correlation of histology and ion image in a *P. lactiflora* root cross section at a scanning step size of 10 μm. (**a**) Optical image of a region of interest. (**b–d**) Overlay of optical image and individual ion images including (**a**) *m/z* 519.12632 ([PA/AL + K]^+^), (**b**) *m/z* 979.08134 ([5GG + K]^+^) and (**c**) *m/z* 1435.11422 ([8GG + K]^+^). (**e**) Overlay of ion images for *m/z* 519.12632 (blue, [PA/AL + K]^+^) and *m/z* 979.08134 (red, [5GG + K]^+^). (**f**) Overlay of ion images for 519.12632 (blue, [PA/AL + K]^+^) and *m/z* 1435.11422 (green, [8GG + K]^+^). (**g**) Overlay of ion images for *m/z* 979.08134 (red, [5GG + K]^+^) and *m/z* 1435.11422 (green, [8GG + K]^+^). The regions presenting subtle differences in xylem were marked with a white line. All ion images were generated with a bin width of ± 5 ppm.

**Figure 7:**
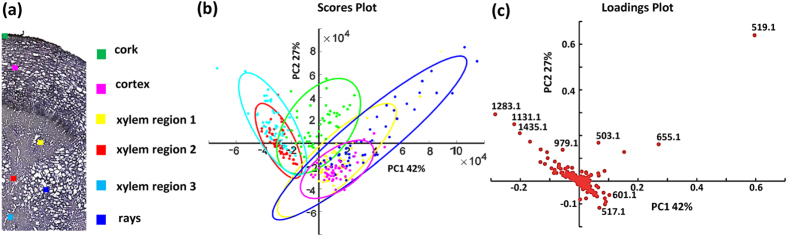
PCA of 10 μm AP-MALDI MSI data from *P. lactiflora* root section. (**a**) Optical image and 6 regions selected for PCA. (**b**) PCA derived from intensities of 6 regions in AP-MALDI MSI. (**c**) PC loadings derived from all *m/z* peaks of 6 regions in AP-MALDI MSI.

Highly specialized metabolism in plant tissues leads to rather diverse distributions of metabolites. By overlaying optical and ion images (Fig. 6b–d) and individual ion images (Fig. 6e–g) of specific MGs and gallotannins in one composite image, similarities and differences of localization of metabolites become readily evident. As shown in Fig. 6b,c,e, in the cortex, PA/AL and 5GG share similar deposition sites, but in the xylem regions they have complementary distributions, even more pronounced in the composite image of PA/AL (B: blue) and 8GG (G: green) (Fig. 6f). Furthermore, as shown in Figs 3d and 6g, spatial localization of the homologous series of 5GG and complex gallotannins (6GG-9GG) display a rather interesting pattern in which 5GG (known as immediate precursors for the subsequent formation of complex gallotannins or ellagitannins) covers more area in the root tissue section, however, complex tannins are prone to accumulate in well-defined regions i.e. the cortex, xylem vessels and fibres. Additionally, in the cortex region a decreasing trend for the deposition of gallotannins from 5GG to 9GG is resolved as shown in Fig. 3d. With the capability of cellular imaging, subtle differences in vessels and xylem fibres were visualized with high-quality composite ion images (marked with white line in Fig. 6d–g), which may be correlated with the different stages of xylem cell differentiation. Next, mass spectral data of 6 regions of interest, selected from 10 μm and 30 μm ion images, were analyzed by principal component analysis (PCA) (Fig. 7 and Figure S5). Evaluation of PCA of 10 μm ion images allows to differentiate the regions of interest, consistent with high resolution imaging results. The xylem region 2 and 3 showed ion similarities and were totally separated from the xylem region 1 (selected from the region marked with white line in Fig. 6), as well as rays and cortex regions. For xylem region 2 and 3, ions contributing to the highest variance are complex gallotannins including *m/z* 1131.1 ([6GG + K]^+^), 1283.1([7GG + K]^+^) and 1435.1([8GG + K]^+^). For xylem region 1, rays and cortex regions, *m/z* 519.1([PA/AL + K]^+^) played the most significant role in separating from the other cell populations. The cork region was separated from other regions but shared similarities with the other areas. Therefore, the various regions can be distinguished as different groups, and in the xylem vessels and fibres (including xylem region 1, 2, 3) different cell types were found which can possibly be correlated with different stages of cell lignification. However, as shown in Figure S5 these small differences cannot be distinguished in 30 μm measurements but require the higher lateral resolution. In Figure S5, it is possible to see different populations of cells in the loadings plot, but it is apparent that there is significant overlap of the scores populations. It should be noted that in instances when the ionization of target analytes are influenced by endogenous chemical components, matrix ions, or different physical properties of the plant tissues the correlation between signal intensity and concentration is reduced. In particular, for plant tissues they impact on the lower limits of detection of the surface analytes, signal stability and reproducibility. All abovementioned issues are significant challenges that need to be overcome in further development of the technology.

Regarding the function of secondary metabolites in ecological and evolutionary processes, the accumulation of gallotannins in particular in the cork and xylem regions (vessels and fibres) of *P. lactiflora* root can possibly be correlated to their great potential as a chemical defence barrier against enemies, as stored in the non-cytoplasmic compartments significantly reduce the risk of harming essential cytoplasmic components^41^. In previous studies, high amounts of tannins, including both condensed and hydrolysable tannins, have been shown to be preferably deposited in the bark area^42^. Moreover, ellagitannins were found to preferentially accumulate in the reaction zone of wood (secondary xylem of woody plants) and may contribute to the effectiveness of the reaction zone as an antimicrobial barrier^43^. Of high interest is the potential interrelationship of lignins and hydrolysable tannins which has been rarely reported^44^. These two classes of components share the general phenylpropanoid pathway for generation of precursors^45^. Xylem cell walls are the major deposition sites of lignins which have been visualized with various microscopy techniques^46^ and ToF-SIMS^47^. Therefore, the high quality ion images of hydrolysable tannins obtained in this study can possibly provide pivotal hints for understanding the physiological role of hydrolysable tannins in *P. lactiflora* root, for example, together with lignins to contribute to the xylem cell functions such as structural support, water conduction, or as a chemical defence barrier.

## Methods

### Chemicals and plant samples

Trifluoroacetic acid (TFA), water (HPLC grade), acetone (HPLC grade) and 2,5-dihydroxybenzoic acid (DHB, 98%) were purchased from Fluka (Neu Ulm, Germany). *Paeonia lactiflora* roots were provided by the Botanical Garden of Justus Liebig University Giessen, Germany.

### Sample preparation

The fresh roots of *P. lactiflora* were collected and stored at −80 °C until use. For cryo-sectioning, the roots were directly fixed on the sample holder of a cryo-microtome (HM 525 cryostat, Thermo Scientific, Dreieich, Germany), using deionized water as the adhesive. Sections of 20 μm thickness were obtained at −18 °C and thaw-mounted on regular glass slides for immediate imaging measurements or stored at −80 °C until analysis. To avoid condensation, tissue sections were dehydrated in a vacuum desiccator for ca. 5 minutes prior to matrix application. A dedicated pneumatic sprayer (SMALDIPrep, TransMIT GmbH, Giessen, Germany) was used for the uniform application of a DHB matrix solution prepared at concentrations of 30 mg/ml in acetone/water (0.1% TFA) 1:1 v/v. Matrix crystal sizes and uniformity were checked before measurements by visual inspection with an Olympus BX–40 microscope. A uniform matrix layer and crystal sizes <10 μm were obtained, as needed for a MALDI imaging experiment at high spatial resolution (Figure S6). Optical images of tissue sections were acquired before matrix application with the Olympus BX–40 microscope.

### Instrumentation

All measurements were performed using the AP-SMALDI10 high-resolution MALDI imaging ion source (TransMIT GmbH, Giessen, Germany), which was operated at atmospheric pressure and coupled to a Q-Exactive Orbitrap mass spectrometer (Thermo Fisher Scientific, Bremen, Germany). The ion source includes a nitrogen laser (*λ* = 337 nm) operating at a repetition rate of 60 Hz. The laser beam was focused by a centrally bored objective lens to an optical diameter of 8.4 μm (1/e^2^ definition) and an effective ablation spot diameter of 5 μm^48^. For experiments at 30 μm pixel size, the laser was slightly defocused, and the laser energy was correspondingly increased in order to increase the irradiation area and thus the ion yield. For imaging at 10 μm pixel size an ablation spot diameter of 5 μm was used. For each mass spectrum, ions from 30 laser pulses were accumulated in the C-trap before being sent to the Orbitrap mass analyser. All experiments were performed in positive-ion mode with the target voltage set to + 4.3 kV.

Mass spectrometry imaging was performed in two adjacent sections at 30 μm and 10 μm spatial resolution in an area of 7800 × 4200 μm^2^ (260 × 140 pixels) and 3500 × 1400 μm^2^ (360 × 140 pixels) respectively. The measurement speed in full scan mode (scan range *m/z* 400–1600) was about 1.3 seconds per pixel at a mass resolution of 140,000 @ *m/z* 200). The step size of the sample stage was set to the desired pixel size. Internal lock-mass calibration was used, providing a mass accuracy of better than 3 ppm RMSE over the course of the entire run. MS/MS measurements for compound identification were performed by line scanning over the sample.

### Data processing

Selected ion images were generated with the imaging software package MIRION^49^. All images were generated with a mass bin width of *m/z* ± 5 ppm from the exact mass. Single ion images were normalized to the highest intensity measured for each ion separately. RGB images were obtained by selecting three different mass signals for the three red-green-blue channels. The accurate mass (*m*_*c*_) of ions was used for image generation, mass accuracy and root mean square error (RMSE) were calculated by using formula (i), (ii) and (iii) respectively, described in supplementary method S1. Overlaid optical and ion images were generated with the open-source software MSiReader v0.06^50^. All images were generated using a bin width of ± 5 ppm without any normalization or interpolation.

Principal component analysis (PCA) and image similarity of paeoniflorin and its derivatives was calculated using MATLAB™ software (Mathworks, Inc., Sherborn, MA, USA and MATLAB™ (The MathWorks GmbH, Ismaning, Germany). Using MSiReader, six regions of interest determined from the optical image and ion images were extracted, producing a text file containing each pixel and its respective spectra between *m/z* 400–1600 binned at *m/z* ± 0.1. The number of pixels selected was n = 50 for 10 μm and n = 16 for 30 μm. Due to the scale of the image, the area had to be kept constant for an accurate evaluation. Using home-written MATLAB code in combination with a confidence ellipse (Douglas M. Schwarz), PCA plots with a 95% confidence ellipse were produced for each region of interest.

## Additional Information

**How to cite this article**: Li, B. *et al*. High-resolution MALDI mass spectrometry imaging of gallotannins and monoterpene glucosides in the root of *Paeonia lactiflora.* *Sci. Rep.* **6**, 36074; doi: 10.1038/srep36074 (2016).

**Publisher’s note:** Springer Nature remains neutral with regard to jurisdictional claims in published maps and institutional affiliations.

## Supplementary Material

Supplementary Information

## Figures and Tables

**Table 1:** Selected gallotannins assigned in *P. lactiflora* root tissues by AP-SMALDI-MSI.

Compound^a^	Molecular formula	Adduct	Exact mass (u)	30 μm		10 μm	
				Mass accuracy (ppm)	Coverage of pixels (%)	Mass accuracy (ppm)	Coverage of pixels (%)
pentagalloylglucose (5GG)	C_41_H_32_O_26_	[M + K]^+^	979.08134	−0.38	73.5	−0.63	90.1
hexagalloylglucose (6GG)*	C_48_H_36_O_30_	[M + K]^+^	1131.09230	−0.22	70.3	−0.49	88.4
heptagalloylglucose (7GG)*	C_55_H_40_O_34_	[M + K]^+^	1283.10326	−0.91	51.6	−0.94	68.8
octagalloylglucose (8GG)*	C_62_H_44_O_38_	[M + K]^+^	1435.11422	−1.51	39.1	−1.39	54.6
nonagalloylglucose (9GG)	C_69_H_48_O_42_	[M + K]^+^	1587.12517	−1.21	33.4	−1.23	48.6

^*^Tandem mass spectra were acquired for identification (see Figure S1). The other metabolites were putatively identified based on high mass accuracy of full scan data.

**Table 2:** Selected monoterpene glycosides assigned in *P. lactiflora* root tissues by AP-SMALDI-MSI.

Compound^a^	Molecular formula	Adduct	Exact mass (u)	Mass accuracy (ppm)		RMSE (ppm)	
				30 μm	10 μm	30 μm	10 μm
desbenzoylpaeoniflorin	C_16_H_24_O_10_	[M + K]^+^	415.10011	−0.58	−0.53	0.70	0.63
paeoniflorin/albiflorin	C_23_H_28_O_11_	[M + K]^+^	519.12632	−0.17	0.00	0.36	0.24
oxypaeoniflorin/oxypaeoniflorin isomer	C_23_H_28_O_12_	[M + K]^+^	535.12123	−0.09	0.04	0.51	0.36
galloyl-desbenzoylpaeoniflorin	C_23_H_28_O_14_	[M + K]^+^	567.11106	−0.12	−0.02	0.86	0.68
benzoylpaeoniflorin	C_30_H_32_O_12_	[M + K]^+^	623.15253	−0.26	−0.14	0.93	0.60
galloylpaeoniflorin/galloylalbiflroin	C_30_H_32_O_15_	[M + K]^+^	671.13728	−0.16	−0.07	0.65	0.42
isomaltopaeoniflorin/glucopyranosylalbiflorin	C_29_H_38_O_16_	[M + K]^+^	681.17914	−0.38	−0.29	1.16	0.89

Metabolites were putatively identified based on high mass accuracy of full scan MS data.

**Table 3:** Selected metabolites assigned in *P. lactiflora* root tissues by AP-SMALDI MSI at a 10 μm spatial resolution.

Compound^a^	Molecular formula	Adduct	Exact mass	Mass accuracy (ppm)	RMSE (ppm)
Arginine*	C_6_H_14_N_4_O_10_	[M + H]^+^	175.11895	0.86	0.93
monosaccharide	C_6_H_12_O_6_	[M + K]^+^	219.02655	0.50	0.57
disaccharide	C_12_H_22_O_11_	[M + K]^+^	381.07937	0.00	0.16
trisaccharide*	C_18_H_32_O_16_	[M + K]^+^	543.13219	0.07	0.34
tetrasaccharide*	C_24_H_42_O_21_	[M + K]^+^	705.18502	−0.09	0.47
benzoylsucrose	C_19_H_26_O_12_	[M + K]^+^	485.10558	−0.10	0.70
lactiflorin	C_23_H_26_O_10_	[M + Na]^+^	485.14182	0.02	0.67
pinen-vicianoside	C_21_H_34_O_10_	[M + K]^+^	485.17836	−0.14	0.69
glucopyranosyl-enzoylpaeonisuffrone	C_23_H_28_O_10_	[M + Na]^+^	487.15747	0.12	0.95
galloylsucrose	C_19_H_26_O_15_	[M + K]^+^	533.09033	0.21	0.99
mudanpioside E	C_24_H_30_O_13_	[M + K]^+^	565.13180	−0.04	0.69
di-O-galloylpaeoniflorin	C_37_H_36_O_19_	[M + K]^+^	823.14824	−0.06	0.85
PC (34:2)*	C_42_H_80_NO_8_P	[M + K]^+^	796.52531	−0.19	0.31
PC (36:4)*	C_44_H_80_NO_8_P	[M + K]^+^	820.52531	−0.12	0.33

^*^Tandem mass spectra were acquired for identification (see Figure S1). The other metabolites were putatively identified based on high mass accuracy of full scan data.
