# Supplementary material for: High-resolution MALDI mass spectrometry imaging of gallotannins and monoterpene glucosides in the root of Paeonia lactiflora
Source: Sci Rep. 2016 Oct 31;6:36074. doi: 10.1038/srep36074 (PMC5086847; doi:10.1038/srep36074)

**Supplementary material**

**High-resolution MALDI mass spectrometry imaging of gallotannins and monoterpene glucosides in the root of *Paeonia lactiflora***

Bin Li^1,2,3†^, Dhaka Ram Bhandari^1†^, Andreas Römpp^1,4^, Bernhard Spengler^1^*

^1^Institute of Inorganic and Analytical Chemistry, Justus Liebig University Giessen, Schubertstrasse 60, building 16, 35392 Giessen, Germany,

^2^Department of Pharmacy, University of Copenhagen, Universitetsparken 2, 2100, Copenhagen, Denmark.

^3^Now: Department of Chemistry and Beckman Institute for Advanced Science and Technology, University of Illinois at Urbana-Champaign, 61801, Urbana, IL, USA

^4^Now: Department of Food Analysis, University of Bayreuth, E.-C.-Baumann-Str. 20, 95326 Kulmbach, Bayreuth, Germany

*Corresponding author: [Bernhard.Spengler@anorg.Chemie.uni-giessen.de](mailto:Bernhard.Spengler@anorg.Chemie.uni-giessen.de)

**
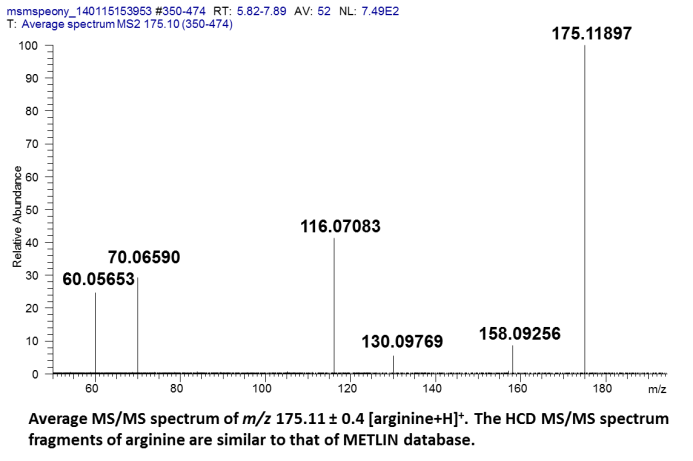

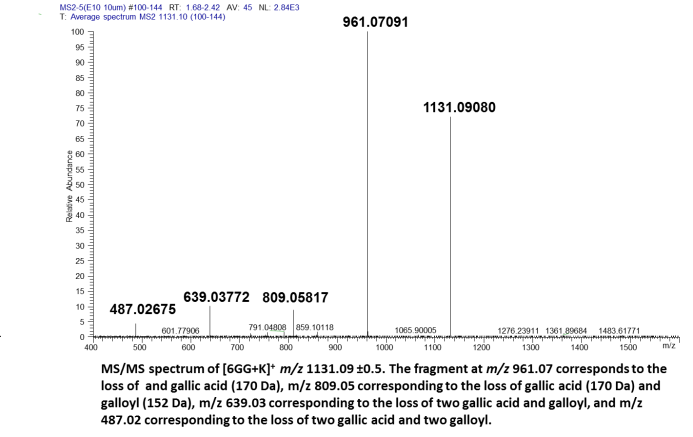
**

**
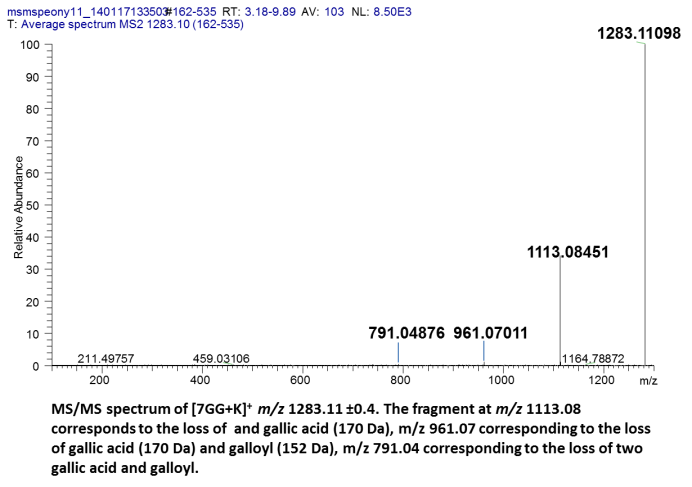

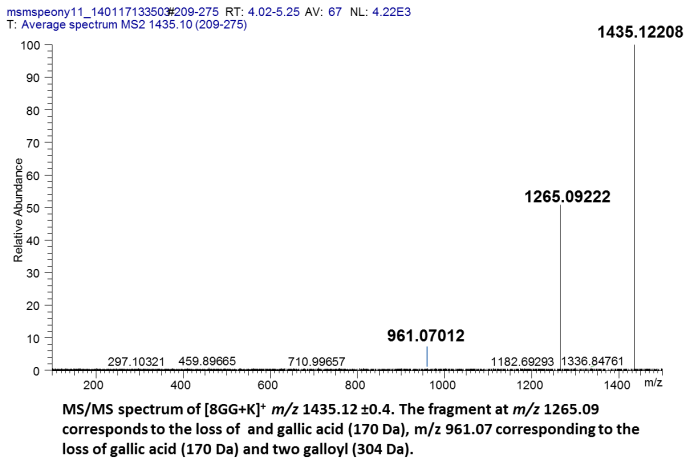
**

**
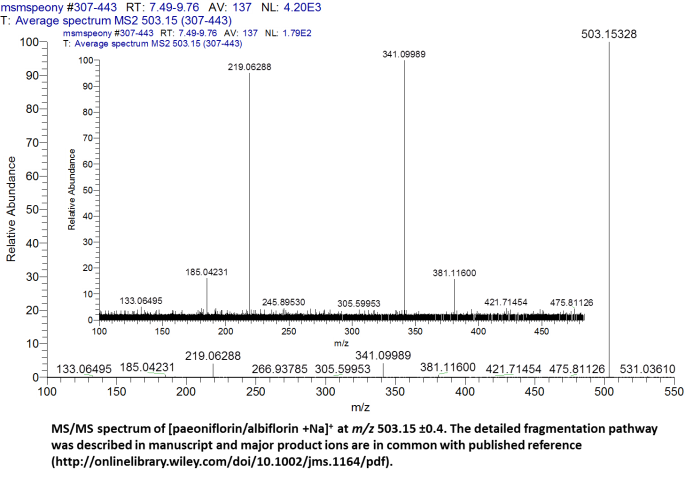

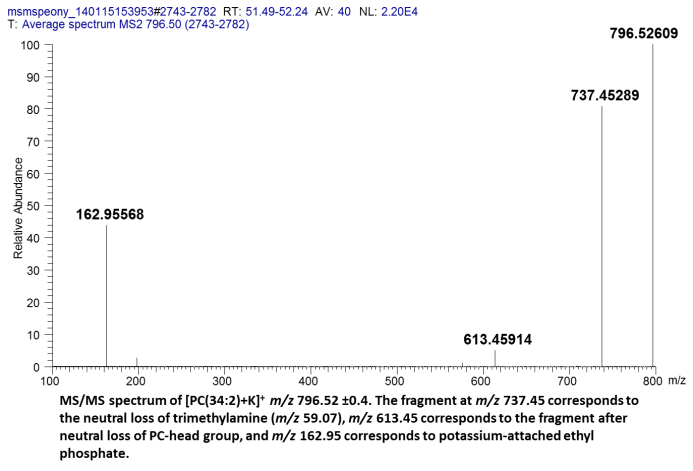
**

**
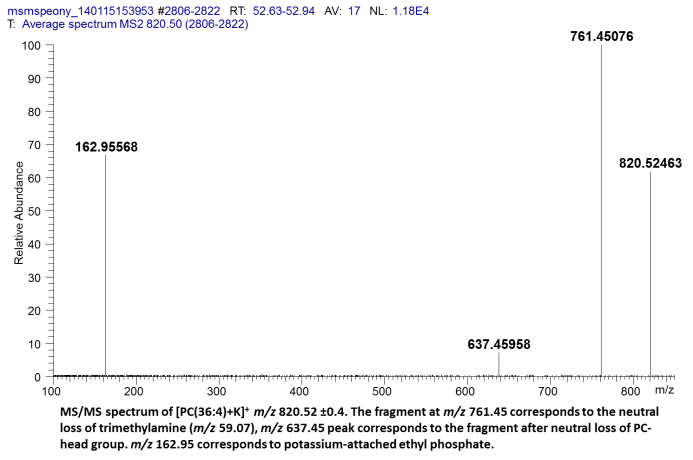

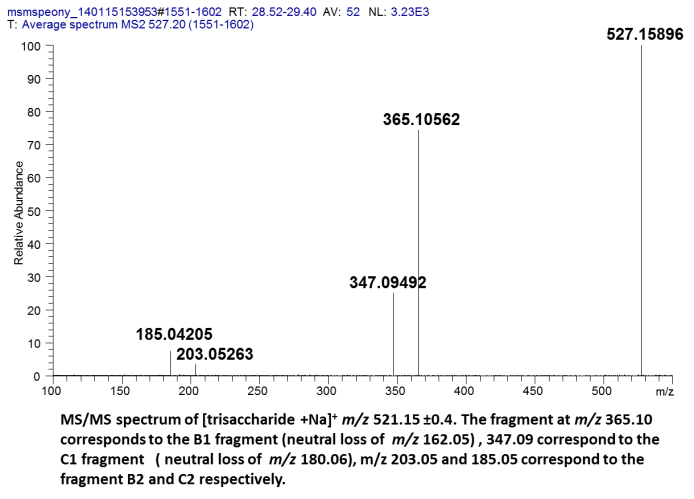
**

**
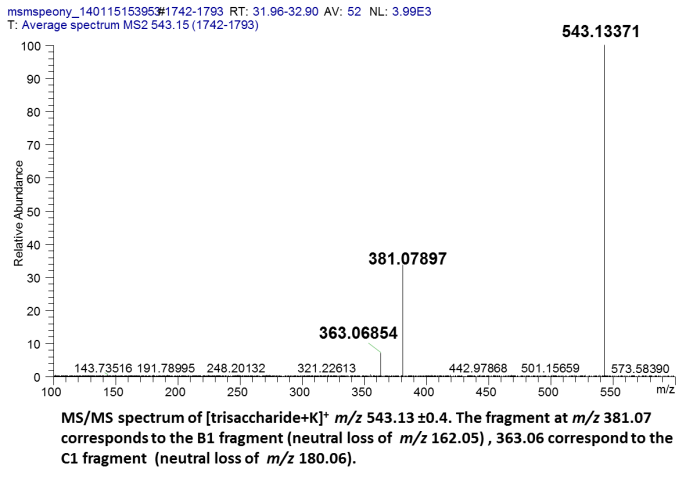

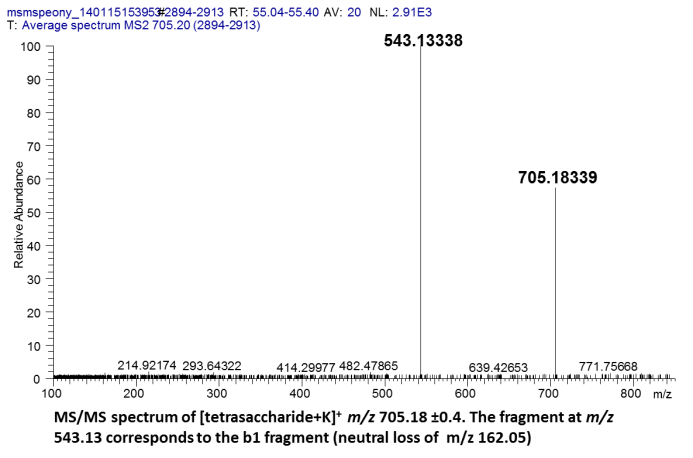
**

**Figure S1.** On-tissue tandem mass spectra of selected compounds.

**
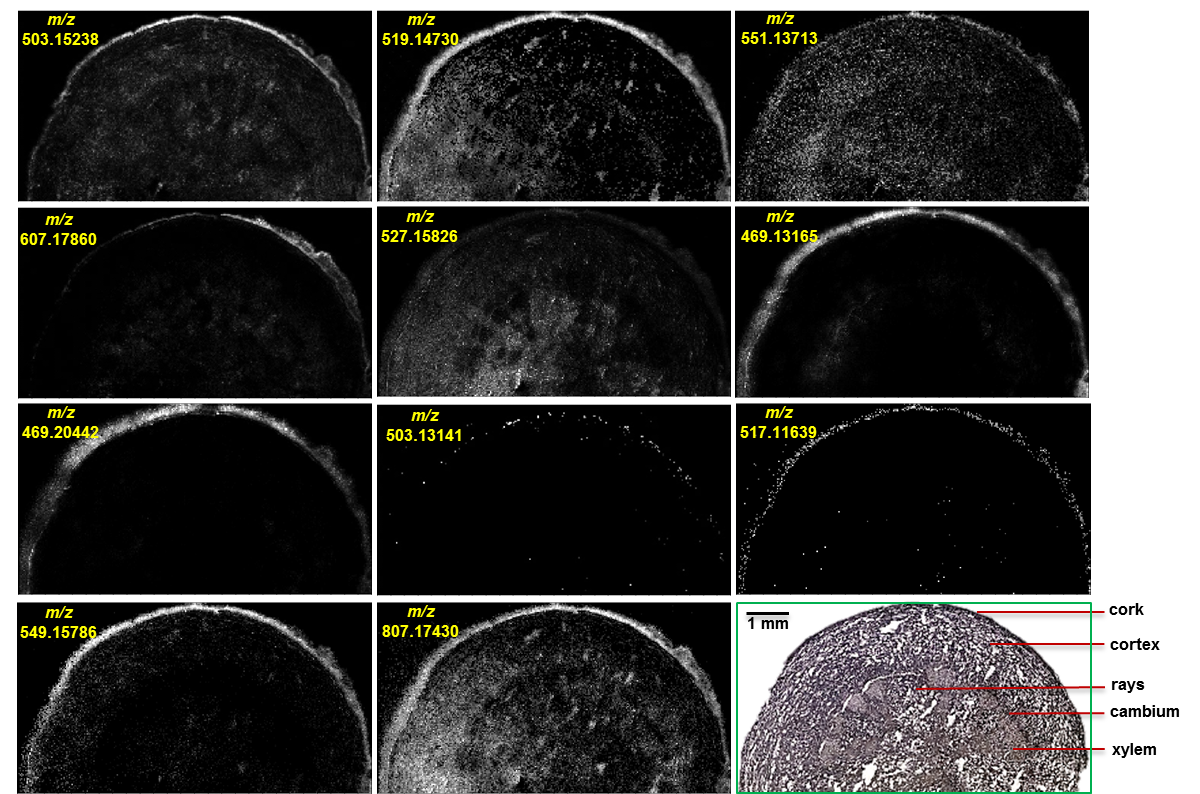
**

**Figure S2.** MALDI images of selected metabolites in the *P. lactiflora* root recorded with a pixel size of 30 µm. All ions are displayed using the same intensity scale (Gray: 0-255). The mass accuracy was better than 3 ppm (RMSE) and a bin width of *m/z* = ± 5 ppm was used. Each individual image represents the K^+^/Na^+^ adducts of the compounds included in **Table S1**.

**
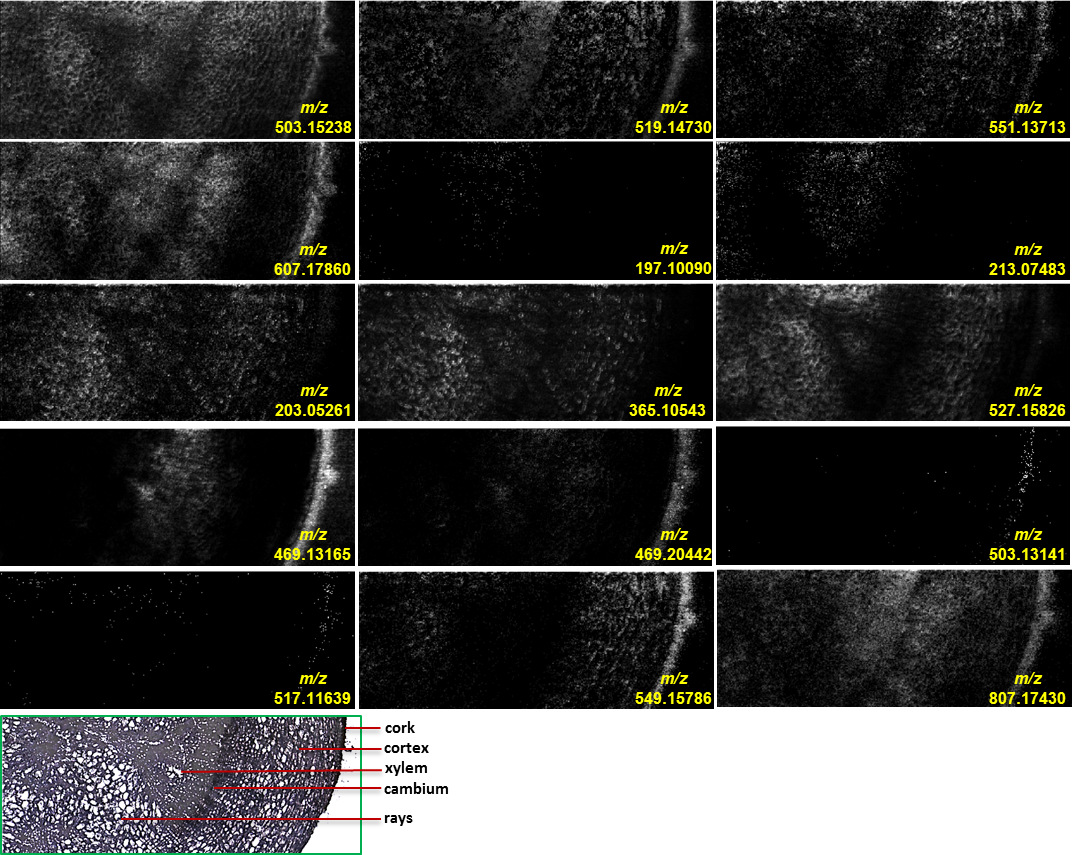
**

**Figure S3.** MALDI images of selected metabolites in the *P. lactiflora* root recorded with a pixel size of 10 µm. All ions are displayed using the same intensity scale (Gray: 0-255). The mass accuracy was better than 3 ppm (RMSE) and a bin width of *m/z* = ± 5 ppm was used. Each individual image represents the K^+^/Na^+^ adducts of the compounds included in **Table S1**.

**
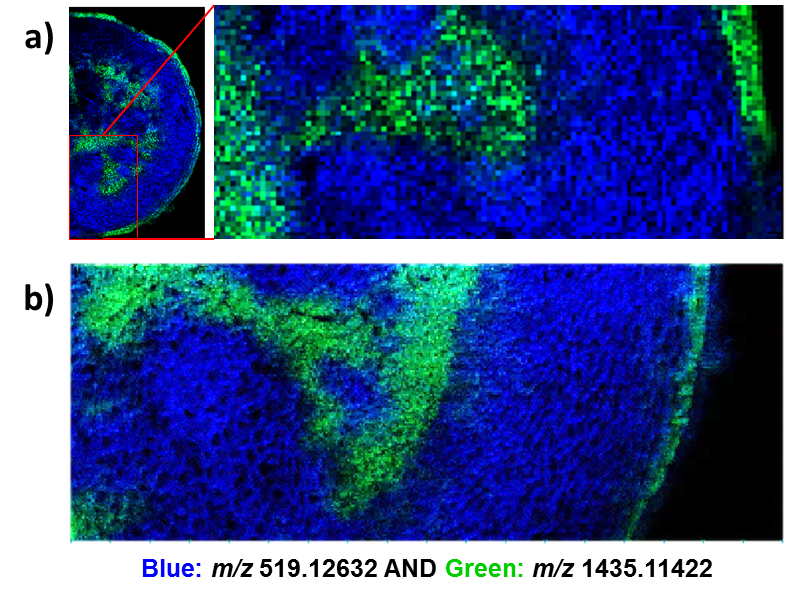
**

**Figure S4.** Overlaid ion images in a *P. lactiflora* root cross section at a scanning step size of a) 30 µm and b) 10 µm, respectively. a) Overlay of ion images for 519.12632 (blue, [PA/AL + K]^+^) and *m/z* 1435.11223 (green, [8GG + K]^+^), recorded with a pixel size of 30 µm. b) Overlay of ion images for *m/z* 979.08134 (red, [5GG+K]^+^) and *m/z* 1435.11223 (green, [8GG + K]^+^), recorded with a pixel size of 10 µm. The ion images were generated with a bin width of ±5 ppm.


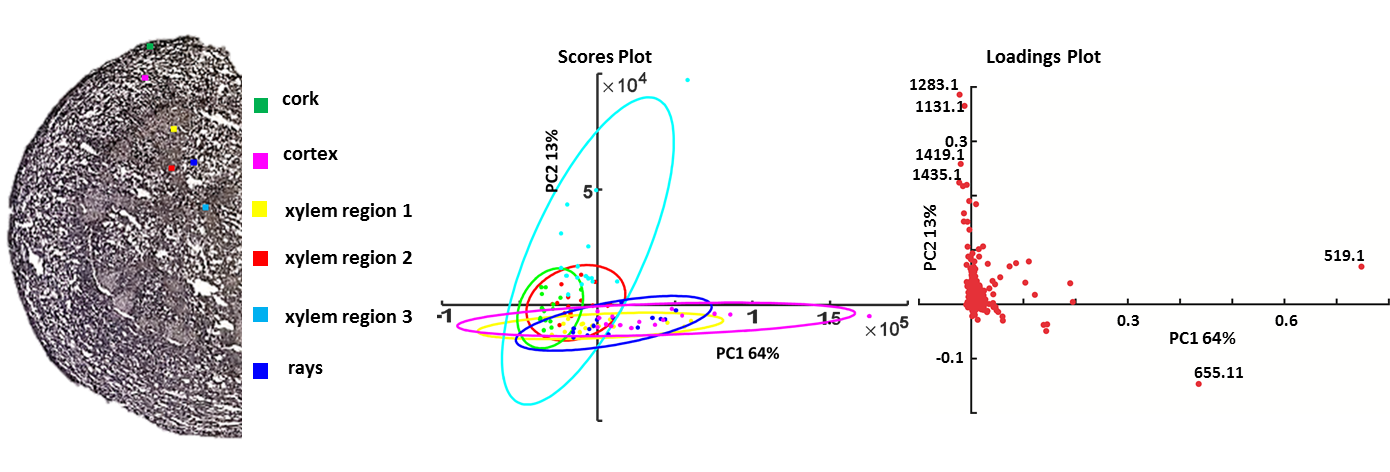


**Figure S5**. PCA of 30 µm AP-MALDI MSI data from *P. lactiflora* root section. (a) Optical image and 6 regions selected for PCA. (b) PCA derived from intensities of 6 regions in AP-MALDI MSI. (c) PC loadings derived from all m/z peaks of 6 regions in AP-MALDI MSI.

**
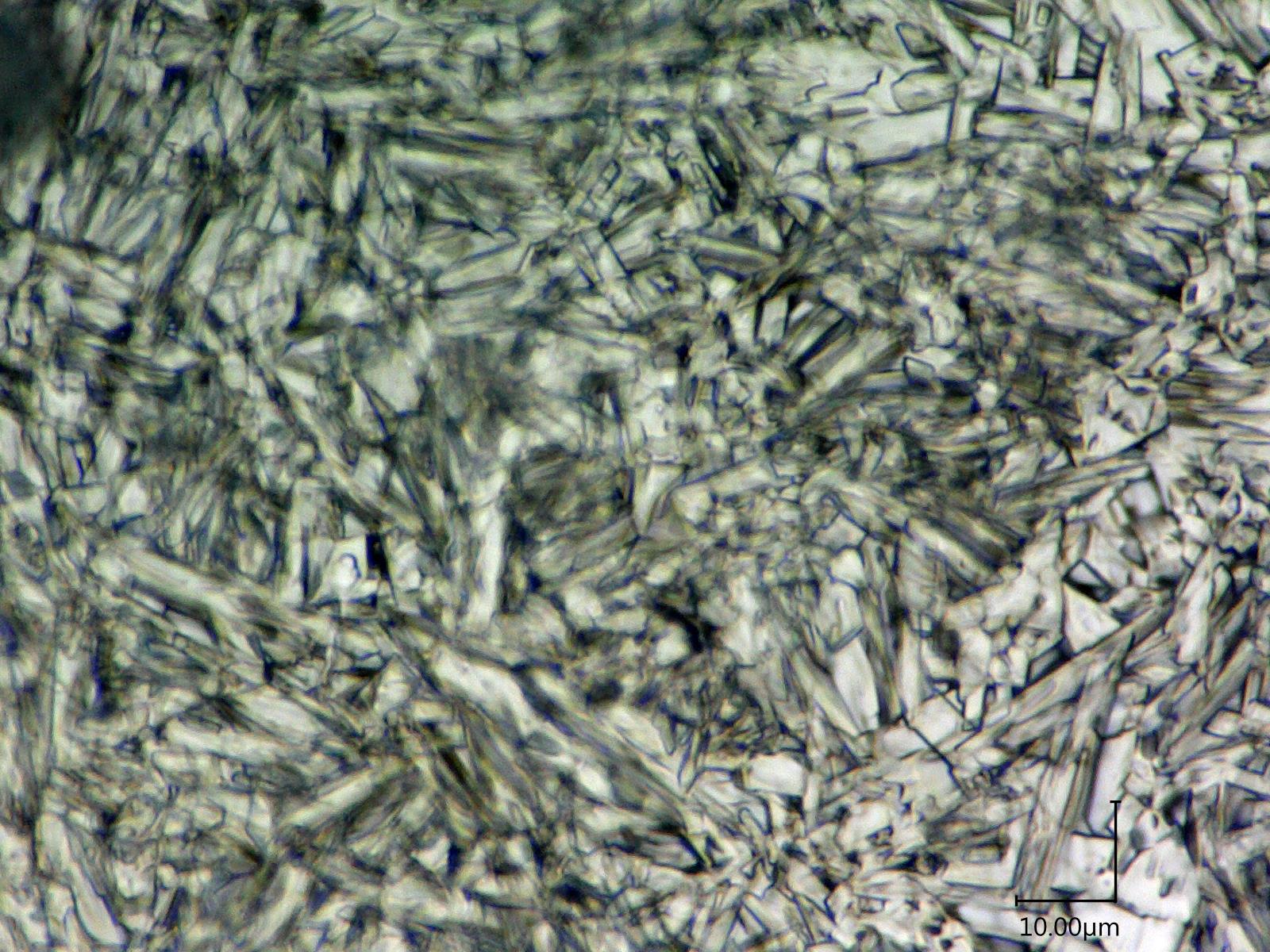
**

**Figure S6.** Microscopic optical image of DHB crystals deposited onto sample surface by a dedicated pneumatic sprayer (SMALDIPrep, TransMIT GmbH, Giessen, Germany). Image was obtained using a Keyence VHX-5000 digital microscope (Keyence Deutschland GmbH)

**Figure S7.** Representative mass spectrum from single blank pixel.

**Table S1. Selected metabolites assigned in *P. lactiflora* root tissues by AP-SMALDI MSI.**

| **Compounds** | **Molecular formula** | **Adduct** | **Exact mass** | **30 µm measurment** | | | | **10 µm measurement** | | | |
| --- | --- | --- | --- | --- | --- | --- | --- | --- | --- | --- | --- |
|  |  |  |  | **Accurate mass** | **Mass accuracy (ppm)** | **RMSE(ppm)** | **Number of pixels** | **Accurate mass** | **Mass accuracy (ppm)** | **RMSE**  **(ppm)** | **Number of pixels** |
| **pentagalloylglucose** | C_41_H_32_O_26_ | [M+K]^+^ | 979.08134 | 979.08097 | -0.38 | 0.99 | 26760 | 979.08072 | -0.63 | 0.85 | 45423 |
| **hexagalloylglucose** | C_48_H_36_O_30_ | [M+K]^+^ | 1131.09230 | 1131.09205 | -0.22 | 0.88 | 25600 | 1131.09175 | -0.49 | 0.76 | 44569 |
| **heptagalloylglucose** | C_55_H_40_O_34_ | [M+K]^+^ | 1283.10326 | 1283.10209 | -0.91 | 1.84 | 18796 | 1283.10206 | -0.94 | 1.77 | 34659 |
| **octagalloylglucose** | C_62_H_44_O_38_ | [M+K]^+^ | 1435.11422 | 1435.11206 | -1.51 | 2.12 | 14241 | 1435.11223 | -1.39 | 2.27 | 27535 |
| **nonagalloylglucose** | C_69_H_48_O_42_ | [M+K]^+^ | 1587.12517 | 1587.12325 | -1.21 | 1.84 | 12166 | 1587.12321 | -1.23 | 1.76 | 24483 |
|  |  |  |  |  |  |  |  |  |  |  |  |
| **desbenzoylpaeoniflorin** | C_16_H_24_O_10_ | [M+Na]+ | 399.12617 | / | / | / | / | 399.12622 | 0.13 | 0.77 | 531 |
| **desbenzoylpaeoniflorin** | C_16_H_24_O_10_ | [M+K]+ | 415.10011 | 415.09987 | -0.58 | 0.70 | 28886 | 415.09989 | -0.53 | 0.63 | 44074 |
| **paeoniflorin /albiflorin** | C_23_H_28_O_11_ | [M+Na]+ | 503.15238 | 503.15215 | -0.46 | 0.61 | 29738 | 503.15225 | -0.26 | 0.34 | 49937 |
| **paeoniflorin /albiflorin** | C_23_H_28_O_11_ | [M+K]+ | 519.12632 | 519.12623 | -0.17 | 0.36 | 28736 | 519.12632 | 0.00 | 0.24 | 47067 |
| **oxypaeoniflorin /oxypaeoniflorin isomer** | C_23_H_28_O_12_ | [M+Na]+ | 519.14730 | 519.14639 | -1.75 | 2.20 | 13782 | 519.14627 | -1.98 | 2.13 | 22215 |
| **oxypaeoniflorin /oxypaeoniflorin isomer** | C_23_H_28_O_12_ | [M+K]+ | 535.12123 | 535.12118 | -0.09 | 0.51 | 28459 | 535.12125 | 0.04 | 0.36 | 45310 |
| **6'-O-galloyl desbenzoylpaeoniflorin** | C_23_H_28_O_14_ | [M+Na]+ | 551.13713 | 551.13696 | -0.31 | 1.43 | 16130 | 551.13705 | -0.15 | 1.17 | 12702 |
| **6'-O-galloyl desbenzoylpaeoniflorin** | C_23_H_28_O_14_ | [M+K]+ | 567.11106 | 567.11099 | -0.12 | 0.86 | 22098 | 567.11105 | -0.02 | 0.68 | 32816 |
| **benzoylpaeoniflorin** | C_30_H_32_O_12_ | [M+Na]+ | 607.17860 | 607.17845 | -0.25 | 0.91 | 18518 | 607.17858 | -0.03 | 0.70 | 38498 |
| **benzoylpaeoniflorin** | C_30_H_32_O_12_ | [M+K]+ | 623.15253 | 623.15237 | -0.26 | 0.93 | 21598 | 623.15244 | -0.14 | 0.60 | 43341 |
| **benzoyloxypaeoniflorin/albiflorinR3** | C_30_H_32_O_13_ | [M+Na]+ | 623.17351 | 623.17346 | -0.08 | 1.53 | 13617 | 623.17337 | -0.22 | 0.96 | 20919 |
| **galloylpaeoniflorin/ galloylalbiflroin** | C_30_H_32_O_15_ | [M+K]+ | 671.13728 | 671.13717 | -0.16 | 0.65 | 28140 | 671.13723 | -0.07 | 0.42 | 45417 |
|  |  |  |  |  |  |  |  |  |  |  |  |
| **arginine** | C_6_H_14_N_4_O_2_ | [M+H]^+^ | 175.11895 | / | / | / | / | 175.11910 | 0.86 | 0.93 | 19059 |
| **arginine** | C_6_H_14_N_4_O_2_ | [M+Na]^+^ | 197.10090 | / | / | / | / | 197.10105 | 0.76 | 1.10 | 606 |
| **arginine** | C_6_H_14_N_4_O_2_ | [M+K]^+^ | 213.07483 | / | / | / | / | 213.07494 | 0.52 | 0.82 | 2761 |
| **monosaccharide** | C_6_H_12_O_6_ | [M+Na]^+^ | 203.05261 | / | / | / | / | 203.05272 | 0.54 | 0.68 | 31267 |
| **monosaccharide** | C_6_H_12_O_6_ | [M+K]^+^ | 219.02655 | / | / | / | / | 219.02666 | 0.50 | 0.57 | 43301 |
| **disaccharide** | C_12_H_22_O_11_ | [M+Na]^+^ | 365.10543 | / | / | / | / | 365.10548 | 0.14 | 0.28 | 45713 |
| **disaccharide** | C_12_H_22_O_11_ | [M+K]^+^ | 381.07937 | / | / | / | / | 381.07937 | 0.00 | 0.16 | 44411 |
| **trisaccharide** | C_18_H_32_O_16_ | [M+Na]^+^ | 527.15826 | 527.15813 | -0.25 | 0.45 | 30390 | 527.15823 | -0.06 | 0.39 | 47296 |
| **trisaccharide** | C_18_H_32_O_16_ | [M+K]^+^ | 543.13219 | 543.13216 | -0.06 | 0.48 | 29200 | 543.13223 | 0.07 | 0.34 | 45154 |
| **tetrasaccharide** | C_24_H_42_O_21_ | [M+K]^+^ | 705.18502 | 705.18491 | -0.16 | 0.50 | 29107 | 705.18496 | -0.09 | 0.47 | 45021 |
| **1´-O-benzoylsucrose** | C_19_H_26_O_12_ | [M+Na]^+^ | 469.13165 | 469.13156 | -0.19 | 1.01 | 22175 | 469.13157 | -0.17 | 0.64 | 27545 |
| **1´-O-benzoylsucrose** | C_19_H_26_O_12_ | [M+K]^+^ | 485.10558 | 485.10561 | 0.06 | 1.01 | 24706 | 485.10553 | -0.10 | 0.70 | 30256 |
| **lactiflorin** | C_23_H_26_O_10_ | [M+Na]^+^ | 485.14182 | 485.14170 | -0.25 | 0.88 | 26161 | 485.14183 | 0.02 | 0.67 | 31512 |
| **ß-pinen-10-yl-ß-vicianoside** | C_21_H_34_O_10_ | [M+Na]^+^ | 469.20442 | 469.20419 | -0.49 | 0.87 | 12617 | 469.20431 | -0.23 | 0.73 | 16874 |
| **ß-pinen-10-yl-ß-vicianoside** | C_21_H_34_O_10_ | [M+K]^+^ | 485.17836 | 485.17821 | -0.31 | 0.93 | 17736 | 485.17829 | -0.14 | 0.69 | 28089 |
| **1-O-ß-D-glucopyranosyl-8-O-benzoylpaeonisuffrone** | C_23_H_28_O_10_ | [M+Na]^+^ | 487.15747 | 487.15724 | -0.47 | 1.34 | 10026 | 487.15753 | 0.12 | 0.95 | 9764 |
| **1-O-ß-D-glucopyranosyl-8-O-benzoylpaeonisuffrone** | C_23_H_28_O_10_ | [M+K]^+^ | 503.13141 | 503.13170 | 0.58 | 2.30 | 145 | 503.13179 | 0.76 | 2.27 | 234 |
| **galloylsucrose** | C_19_H_26_O_15_ | [M+Na]^+^ | 517.11639 | 517.11591 | -0.93 | 1.69 | 680 | 517.11631 | -0.15 | 1.44 | 258 |
| **galloylsucrose** | C_19_H_26_O_15_ | [M+K]^+^ | 533.09033 | 533.09040 | 0.13 | 0.87 | 11710 | 533.09044 | 0.21 | 0.99 | 6993 |
| **mudanpioside E** | C_24_H_30_O_13_ | [M+Na]^+^ | 549.15786 | 549.15754 | -0.58 | 1.45 | 10348 | 549.15777 | -0.16 | 0.92 | 13488 |
| **mudanpioside E** | C_24_H_30_O_13_ | [M+K]^+^ | 565.13180 | 565.13169 | -0.19 | 0.87 | 21429 | 565.13178 | -0.04 | 0.69 | 28173 |
| **3',6'-di-O-galloylpaeoniflorin** | C_37_H_36_O_19_ | [M+Na]^+^ | 807.17430 | 807.17405 | -0.31 | 1.28 | 22364 | 807.17365 | -0.81 | 1.66 | 31858 |
| **3',6'-di-O-galloylpaeoniflorin** | C_37_H_36_O_19_ | [M+K]^+^ | 823.14824 | 823.14826 | 0.02 | 1.19 | 30090 | 823.14819 | -0.06 | 0.85 | 43140 |
| **PC(34:2)** | C_42_H_80_NO_8_P | [M+K]^+^ | 796.52531 | 796.52517 | -0.18 | 0.33 | 27936 | 796.52516 | -0.19 | 0.31 | 45320 |
| **PC(36:4)** | C_44_H_80_NO_8_P | [M+K]^+^ | 820.52531 | 820.5252 | -0.13 | 0.35 | 27919 | 820.52521 | -0.12 | 0.33 | 45249 |

**Table S2.** Distribution patterns of paeoniflorin and its derivatives in *P. lactiflora* root tissues.

|  |  |  |  | **Percentage**  **similarity** | |
| --- | --- | --- | --- | --- | --- |
| **Compound** | **Molecular formula** | **Adduct** | **Exact mass** | **30 µm** | **10 µm** |
| **paeoniflorin/albiflorin** | C_23_H_28_O_11_ | [M+K]^+^ | 519.12632 |  |  |
| **desbenzoylpaeoniflorin** | C_16_H_24_O_10_ | [M+K]^+^ | 415.10011 | 85 | 87 |
| **oxypaeoniflorin/oxypaeoniflorin isomer** | C_23_H_28_O_12_ | [M+K]^+^ | 535.12123 | 91 | 94 |
| **galloyl-desbenzoylpaeoniflorin** | C_23_H_28_O_14_ | [M+K]^+^ | 567.11106 | 85 | 88 |
| **benzoylpaeoniflorin** | C_30_H_32_O_12_ | [M+K]^+^ | 623.15253 | 87 | 88 |
| **galloylpaeoniflorin/galloylalbiflroin** | C_30_H_32_O_15_ | [M+K]^+^ | 671.13728 | 88 | 91 |
| **isomaltopaeoniflorin/glucopyranosylalbiflorin** | C_29_H_38_O_16_ | [M+K]^+^ | 681.17914 | 91 | 93 |

Similarity between the images is calculated based on pixel to pixel comparison of *m/z* ion images of paeoniflorin derivatives to *m/z* ion image of paeniflorin images with a minimum brightness of 40 to maximum brightness of 255.

**Supplementary method S1**

**Equation for calculate accurate mass (m_c_,**
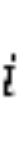
**) of ions used for image generation, mass accuracy (**
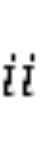
**) and root mean square error (RMSE,**
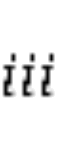
**).**


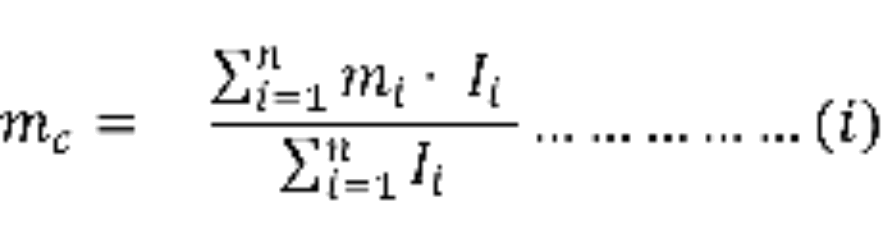


*m_c_* = intensity weighted mean of centroid mass of all the observed masses (m_i_) (accurate mass)

*I* = ion intensity

*m_i_* = centroid mass observed in a single spectrum (single accurate mass)

*n* = number of observations

Mass accuracy in ppm =
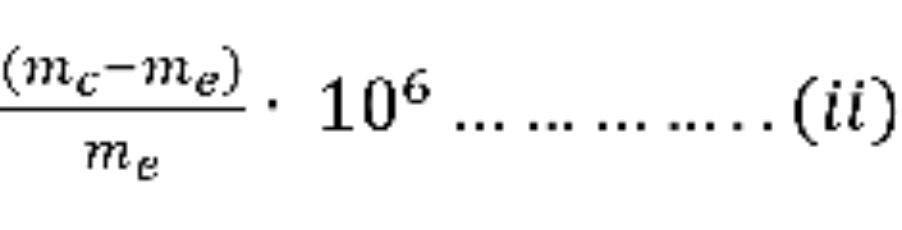


*m_e_* = exact mass (theoretical mass)

Root mean square error (RMSE) =
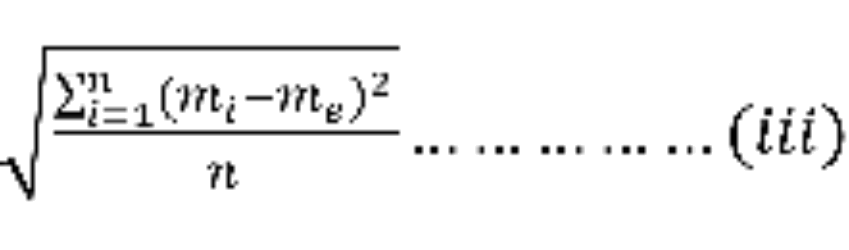

Supplement: Supplementary Information [file srep36074-s1.doc]
